# Identification of Novel Interaction Partners of AIF Protein on the Outer Mitochondrial Membrane

**Published:** 2018

**Authors:** N. P. Fadeeva, N. V. Antipova, V. O. Shender, K. S. Anufrieva, G. A. Stepanov, S. Bastola, M. I. Shakhparonov, M. S. Pavlyukov

**Affiliations:** Shemyakin-Ovchinnikov Institute of Bioorganic Chemistry of the Russian Academy of Sciences, Miklukho-Maklaya Str., 16/10, Moscow, 117997, Russia; Institute of Chemical Biology and Fundamental Medicine, Siberian Branch, Russian Academy of Sciences, Akad. Lavrentiev Ave., 8, Novosibirsk, 630090, Russia; Department of Neurosurgery, University of Alabama at Birmingham, AL 35294, USA

**Keywords:** AIF, caspase independent apoptosis, glioma, heat-shock proteins

## Abstract

In response to the wide variety of external and internal signals, mammalian cells undergo apoptosis, programmed cell death. Dysregulation of apoptosis is involved in multiple human diseases, including cancer, autoimmunity, and ischemic injuries. Two types of apoptosis have been described: the caspase-dependent one, leading to digestion of cellular proteins, and caspase-independent apoptosis, resulting in DNA fragmentation. The latter type of apoptosis is executed by AIF protein and is believed to have appeared first during evolution. The key step in the caspase-independent apoptosis program is the dissociation of AIF from the outer mitochondrial membrane (OMM). However, the molecular mechanism of interaction between AIF and OMM remains poorly understood. In this study, we demonstrated that AIF can bind to OMM via mortalin protein. We confirmed interaction between AIF and mortalin both *in vitro *and *in vivo *and mapped the amino acid sequences that are important for the binding of these proteins. Next, we showed that apoptosis induction by chemotherapy leads to downregulation of AIF–mortalin interaction and dissociation of AIF from the OMM. Finally, a bioinformatic analysis demonstrated that a high level of mortalin expression correlates with a worse survival prognosis for glioma patients. Altogether, our data revealed that mortalin plays an important role in the regulation of the caspase-independent apoptotic pathway and allowed us to speculate that inhibition of AIF–mortalin interaction may induce a dissociation of AIF from the OMM and subsequent apoptosis of cancer cells.

## INTRODUCTION

Dysregulation of apoptosis is known to be involved in the development of many human diseases. On the one hand, inhibition of apoptosis can promote a malignant transformation of cells [1] and initiation of autoimmune processes [2]. On the other hand, hyperactivation of apoptosis causes the death of normal cells in patients with ischemic injuries (infarction and stroke) [3]. Caspases (cysteine proteases that cleave cellular proteins) have, for a long time, been regarded as the main apoptosis effectors. However, there currently is a growing pool of data attesting to the important role of the caspase-independent apoptotic pathway, with the apoptosis-inducing factor (AIF) playing the major role in this process [4].

Many types of tumors are known to be resistant to the caspase-dependent pathway of cell death; however, all of them are sensitive to AIF-mediated apoptosis [4, 5]. This sensitivity is attributed to the fact that not only is AIF involved in apoptosis, but it is also essential for the functioning of mitochondria. Therefore, any tumor cell contains a significant amount of AIF protein, making it a promising target for the development of anti-tumor drugs [6]. On the other hand, AIF plays a crucial role in ischemic injuries to healthy cells. Recent studies have shown that the death of muscle and nerve cells in infarctions [7] and strokes [8] occurs only via caspase-independent AIF-mediated apoptosis, while inhibition of AIF before simulation of an infarction model efficiently prevents cell death, making it possible to completely restore the cardiac function two weeks after the simulated infarction [7].

The AIF protein encoded by the *aifm1 *gene is a NAD-binding flavoprotein that normally localizes on the mitochondrial membrane. This protein is synthesized as a 67 kDa pre-propeptide consisting of 613 amino-acid residues (fAIF). Its N-terminus carries the sequence of mitochondrial localization (1–30 a.a.) and the hydrophobic transmembrane segment (66–84 a.a.), followed by two nuclear import sequences, and the FAD- and NAD-binding domains [9, 10]. A comparative analysis of the genomes of different organisms demonstrated that AIF is highly conserved; its close homologs were found in all metazoan organisms, including plants [11]. Interestingly, a loss of this protein causes death at the early stages ontogenesis [12].

AIF synthesized in the cytoplasm is integrated into the inner mitochondrial membrane, subsequently loses its N-terminal segment dueto mitochondrial peptidase activity, and gives rise to 62 kDa mitochondrial AIF (Δ1–54) [10]. During induction of apoptosis, this protein is dissociated from the mitochondrial membrane and transported into the nucleus. In the nucleus, AIF interacts with histone H2AX and inactive CypA endonuclease. The resulting three-component complex cleaves genomic DNA into fragments ~50 kbp long, causing cell death [13].

The kinetics of AIF dissociation from the mitochondrial membrane is very peculiar. According to some findings, this process takes place 12–18 h after induction of apoptosis and results from the activation of the conventional caspase-dependent apoptotic pathway [14]. However, AIF translocation was observed in other studies as soon as 10–20 min after the induction of apoptosis, much earlier than activation of the caspase-dependent pathway takes place [15]. Such contradictory results are attributed to the fact that two pools of AIF exist inside the cell. One fraction of AIF (~70%) is anchored to the inner mitochondrial membrane through the AIF transmembrane segment. Dissociation of these molecules starts when the integrity of the OMM is disrupted, and AIF is cleaved between the amino acids 96 and 120, which results in the formation of soluble Δ1– 102 (57 kDa) or Δ1–118 (55 kDa) AIF fragments [10]. On the other hand, approximately 30% of AIF localizes on the cytoplasmic surface of the outer mitochondrial membrane (OMM). Binding between these molecules and the membrane is much weaker; so, alteration of AIF conformation caused by its interaction with poly-ADP-ribose synthesized in response to DNA damage is enough to trigger AIF dissociation [8, 16].

It is assumed that the highly mobile fraction of AIF anchored to the OMM is responsible for the caspase-independent apoptotic pathway in malignant and normal cells. However, it remains unclear how AIF and OMM interact and why dissociation of this protein takes place. In order to answer this question, we have identified proteins capable of forming a complex with AIF on the OMM and studied how induction of apoptosis alters the interaction between AIF and these proteins.

## MATERIALS AND METHODS

**Purification of outer mitochondrial membrane proteins**

OMM was purified as described previously [17] with slight modifications. Livers from four mice that were starved for 18 hours were washed with buffer A (70 mM sucrose, 210 mM d-mannitol, 0.1 mM EDTA, 1 mM Tris-HCl, pH 7.2), cut into 2- to 4-mm pieces and homogenized with Dounce homogenizer in a 10x volume of buffer A on ice. Next, the solution was centrifuged for 10 min at 500g. Supernatant was collected and centrifuged for 10 min at 9000g. Supernatant was decanted, and the surface of the pallet was carefully washed three times with a small amount of buffer A. Next, pellet was re-suspended in 35 ml of buffer D (20 mM Na-phosphate, 0.02% BSA, pH 7.2) and incubated 20 min on ice. The solution was centrifuged for 20 min at 35000g. Supernatant was decanted, and the pellet was re-suspended in 35 ml of buffer D and centrifuged for 15 min at 1900g. Next, the supernatant was carefully removed and centrifuged for 20 min at 35000g. The yellow-brownish pellet obtained after centrifugation represented a purified OMM fraction. The OMM proteins were solubilized in PBS containing 1% TritonX100, 0.1% Na-deoxycholate and 0.5 mM DTT.

**Plasmid Construction**

The DNA fragment encoding Mortalin was amplified from U87MG cDNA by the PCR technique using the primer pair Mort_for (AAAA AGA TCT ATG ATA AGT GCC AGC CGA GC) and Mort_rev (ACCA GTC GAC CTG TTT TCT CCT TTT GAT) and cloned into the BglIII/SalI sites of the pET28a+ plasmid (Novagen) to generate the pET28-Mort_FULL plasmid. The DNA fragment encoding the N-terminal domain of Mortalin was amplified from pET28-Mort_FULL plasmid by the PCR technique using the primer pair Mort_for and Mort_I_rev (AATA GTC GAC TCA GCC GGC CAA CAC ACC TC) and cloned into the BglIII/SalI sites of the pET28a+ plasmid to generate the pET28-Mort_I plasmid. The DNA fragment encoding the C-terminal domain of Mortalin was amplified from the pET28-Mort_FULL plasmid by the PCR technique using the primer pair Mort_II_for (GGGT AGA TCT ACG GAT GTG CTG CTC) and Mort_rev and cloned into the BglIII/SalI sites of the pET28a+ plasmid to generate the pET28-Mort_II plasmid. For overexpression of full-length AIF fused to Halo-tag, we created the pET-HALO plasmid. The DNA fragment encoding Halo-tag was amplified from the pFC20K HaloTag T7 SP6 Flexi plasmid (Promega) by the PCR technique using the primer pair Halo_for (ACTA ACC GGT CGC CAC CAT GGG ATC CGA AAT CGG TAC TGG) and Halo_rev (AATT AGA TCT ACC GGA AAT CTC CAG AGT A) and cloned into the NcoI/BamHI sites of the pET28a+ plasmid to generate the pET-HALO plasmid. Next, the DNA fragment encoding AIF was amplified from the pEBB-AIF-YC plasmid [18] by the PCR technique using the primer pair AIF_for (AATA GAA TTC GCT AGC TCT GGT GCA TCA GGG G) and AIF_rev (CTGT GTC GAC TCA GTC TTC ATG AAT GTT GA) and cloned into the EcoRI/SalI sites of the pET-HALO plasmid to generate the pET-HALO-AIF plasmid. For overexpression of AIF fused to His-tag, we digested the pET-HALO-AIF plasmid with EcoRI/SalI restriction endonucleases and the resulting DNA fragments were cloned into the corresponding sites of the pet28a+ plasmid to generate the pET28-AIF_FULL plasmid. The DNA fragment encoding apoAIF was amplified from the pEBB-AIF-YC plasmid by the PCR technique using the primer pair apoAIF_for (TAGA GAA TTC GGG CTG ACA CCA GAA CAG A) and AIF_rev and cloned into the EcoRI/SalI sites of the pet28a+ plasmid to generate the pET28-apoAIF plasmid. For overexpression of Mortalin fused to the N-terminal part of YFP, we created the pTagYN-N plasmid. The DNA fragment encoding the N-terminal part of YFP was amplified from the pEBB-XIAP-YN plasmid [18] by the PCR technique using the primer pair YN_for (AAAA GTC GAC ATG GTG AGC AAG GGC GAG GAG C) and YN_rev (AAAT GCGG CCGC TCA GGA TCC GCT CAC G) and cloned into the SalI/NotI sites of the pTagCFP-N (Evrogen) plasmid to generate the pTagYN-N plasmid. Next, the pET28-Mort_FULL plasmid was digested with BglII /SalI restriction endonucleases and the resulting DNA fragments were cloned into the corresponding sites of the pTagYN-N plasmid to generate the pTagYN-Mort plasmid. In all cases, the absence of unwanted mutations in the inserts and vector-insert boundaries was verified by sequencing.

**Induction of recombinant protein synthesis in E.coli**

BL21(DE3) Codone^+^ RIL E.coli cells transformed with plasmids and bacteria from a single colony were transferred into 17 ml of a LB medium containing a corresponding antibiotic. After overnight incubation at 37°C, the medium with bacteria was transferred into 200 ml of a fresh LB medium with a corresponding antibiotic. Bacteria were incubated at 37oC on a shaker until OD_600_ reached 0,7. Next, IPTG was added to a final concentration 1mM and bacteria were incubated on the shaker for an additional 18 hours at room temperature.

**Purification of His-tag fusion proteins**

After IPTG induction, 200 ml of the medium with bacteria was centrifuged for 15 min, 5,000 g at 4oC and the pellet was re-suspended in 12 ml of lysis buffer B (pH 8.0, 100 mM NaH_2_PO_4_, 10 mM Tris-HCl, 8 M Urea) and incubated for 1.5 hours at room temperature. Next, the solution was centrifuged for 15 min, 18,000 g at 4oC and supernatant was incubated with 2 ml of Ni-NTA resin for 1 hour under constant agitation. Next, the suspension was transferred to a column and washed with 10 ml of buffer B and 10 ml of buffer C (same as buffer B but pH 6.3). Next, the bounded proteins were eluted with buffer D (buffer C with 250 mM of imidazole) and dialyzed overnight against PBS with 1mM DTT. The purity of the obtained proteins was assessed by electrophoresis and subsequent Coomassie Blue staining.

**Purification of Halo-tag fusion proteins**

After IPTG induction, 200 ml of medium with bacteria was centrifuged for 15 min, 5,000 g at 4oC and the pellet was re-suspended in 6 ml of lysis buffer F (pH 7.9, 50 mM HEPES, 100 mM NaCl, 0.5 mM DTT, 0.5 mM EDTA, 0.005% Igepal and protease inhibitor cocktail). The suspension was sonicated 10 times for 1 min with 2 min resting time between sonications. Some 1.5 ml of the obtained lysate was incubated with 50 μl of Magne HaloTagBeads (Promega) for 1 hour under constant agitation. Next, beads were washed 4 times with buffer F and, after that, equal aliquots of the beads were used for protein interaction assay.

**Recombinant protein pull-down assay**

To obtain a protein complex, 50 μl of the magnetic beads with immobilized Halo-tagged protein was incubated with a His-tagged protein solution. The suspension was incubated overnight at 4oC under constant agitation. Next, the beads were washed 3 times with PBS and the proteins were eluted with SLB buffer (100 mM Tris-HCl pH 6.8, 4% SDS, 5% β- mercaptoethanol, 20% glycerol and 0.02% bromophenol blue).

**Cell lysate protein pull-down assay**

To obtain a protein complex, 50 μl of the magnetic beads with immobilized His-tagged protein was incubated with the lysate prepared from U87MG cells. The suspension was incubated overnight at 4oC under constant agitation. Next, the beads were washed once with mammalian cell lysis buffer (50 mM HEPES pH 7.5, 140 mM NaCl, 1 mM EDTA, 10% glycerol, 1% NP40, 0.1% sodium deoxycholate) and 3 times with PBS. After washing, the proteins were eluted with PBS containing 250 mM of imidazole.

**Immunoblotting**

Immunoblotting was performed as described previously [19]. The following antibodies were used: anti-AIF 1 : 500 (ab32516, Abcam), anti-Mortalin 1 : 250 (sc133137, Santa Cruz), HRP- conjugated secondary antibodies against γ-chain of rabbit IgG 1 : 5000 (Sigma) and HRP- conjugated secondary antibodies against mouse IgG (Sigma).

**Cell Culture**

Cells were grown in air enriched with 5% (v/v) CO_2_ at 37°C in Dulbecco’s modified Eagle’s medium (DMEM) supplemented with 10% (v/v) fetal bovine serum (FBS), 2mM L-glutamine, and a penicillin (100 units/ml) streptomycin (100μg/ml) mixture. The cells were transfected with a Lipofectamine LTX reagent (Thermo Fisher Scientific; USA) according to the manufacturer’s protocol. Apoptosis was induced by addition of various concentrations of cisplatin (Sigma) or staurosporine (Sigma). Cell viability assay was performed using a Alamar Blue reagent (Thermo Fisher Scientific; USA) according to the manufacturer’s protocol. Briefly, the cells were plated into a 96-well plate (6,000 cells per well). On the next day, cisplatin or staurosporine was added at different concentrations and, 4 days later, cell viability was determined by a Alamar Blue reagent.

**BiFC protein interaction assay**

U87MG cells were plated in the wells of a Lab-Tek II chamber and cotransfected with the pEBB-AIF-YC and pTagYN-N, pTagYN-Mort or pEBB-XIAP-YN plasmids. The next day, the cells were examined with a Leica DM IRE2 confocal microscope. At least 50 cells were analyzed for each plasmid pair.

**PLA assay**

U87MG cells were plated in the wells of a Lab-Tek II chamber and treated with staurosporine for 24 hours. Next, the cells were washed 3 times with phosphate buffered saline (PBS) and fixed with 4% PFA in PBS for 15 min at room temperature. The cells were washed 2 times with PBS and permeabilized with 0.2% Triton-X100 in PBS for 15 min. All subsequent procedures were performed using a Duolink In Situ Orange Starter Kit (Duolink) according to the manufacturer’s protocol.

## RESULTS

**Fig. 1:**
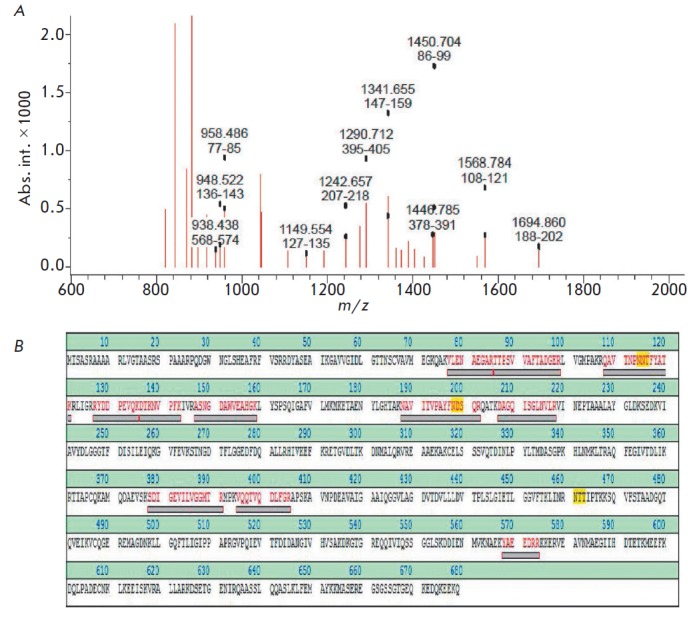
Identification of OMM protein that interacts with full length AIF. A – Mass spectra of OMM proteins that binds to immobilized AIF. Mortalin-related peptides are indicated. B – amino acid sequence of mortalin, peptides that were identified by MALDI-TOF mass spectrometry are highlighted

It was demonstrated earlier that AIF interacts with the OMM surface without being integrated into the lipid bilayer [20]. Taking into account the specificity of AIF binding to the OMM and the fact that this interaction is inhibited by high concentrations of NaCl, we hypothesized that AIF is localized on the OMM surface by binding to a certain adapter protein anchored to the OMM. In order to identify this protein, we studied the interaction between recombinant AIF and proteins isolated from the outer membrane of mouse liver mitochondria by ultracentrifugation. Next, the fraction of solubilized OMM proteins was passed through the sorbent with immobilized control protein or full-length AIF (fAIF). The bound proteins were identified by MALDI-TOF mass spectrometry. One of the proteins capable of interacting with AIF but not with the control recombinant protein was identified as mortalin (*Fig. 1*), a member of the family of heat shock proteins (Hsp) associated with the outer surface of the OMM [21].

**Fig. 2:**
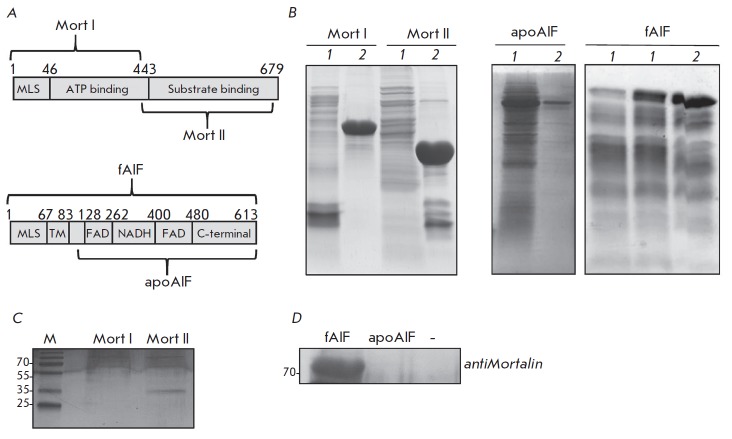
AIF interacts with mortalin *in vitro*. A – schematic representation of mortalin and AIF proteins. The recombinant fragments that were used in this study are indicated. B – electrophoresis of recombinant mortalin and AIF fragments that were purified from E. coli (1 – before optimization of expression and purification conditions; 2 – after optimization). C – electrophoresis demonstrating interaction of mortalin fragments with full-length AIF that was immobilized on magnetic beads. D – Western blotting for endogenous mortalin that was eluted from magnetic beads with immobilized fragments of AIF

Mortalin protein carries two functional domains. They are the ATP-binding domain (1–443 a.a.) at its N-terminus and the peptide-binding domain (444–581 a.a.) at its C-terminus [22]. In order to determine which domain is involved in the interaction with AIF, we tested the binding of fAIF to the N-and C-terminal fragments of mortalin (1–443 a.a. and 433-666, a.a. respectively, *Fig. 2A*). With this aim in mind, we constructed plasmid vectors encoding mortalin fragments which carried a hexahistidine tag at their C-terminus, and a plasmid encoding fAIF with the Halo Tag at its N-terminus. The respective recombinant proteins were isolated from bacterial cells (*Fig. 2B*). Purified mortalin fragments were added to magnetic beads with immobilized fAIF. Following incubation and subsequent washing, solvent-bound proteins were eluted and separated by polyacrylamide gel electrophoresis (*Fig. 2C*). The Mort II fragment interacts with fAIF *in vitro*, while Mort I is incapable of such interaction.

Once the mortalin fragment capable of binding to AIF had been identified, we decided to establish what AIF region is needed for this interaction. For this purpose, we constructed plasmids encoding full-length AIF (fAIF) and processed AIF (apoAIF, amino acids 103 to 613, *Fig. 2A*). Processed AIF emerges upon induction of apoptosis and migrates from the mitochondrial membrane into the cell nucleus after dissociation. Both proteins carried the N-terminal hexahistidine tag. The isolated recombinant AIF fragments were immobilized on magnetic beads. Next, a fraction of solubilized OMM proteins containing mortalin was added to the beads. After incubation and subsequent washing, the bound proteins were eluted and subjected to electrophoresis. Mortalin was detected by Western blot analysis with anti-mortalin primary antibodies. According to the immunodetection data (*Fig. 2D*), endogenous mortalin interacts with full-length recombinant fAIF *in vitro *and does not interact with apoAIF, which is consistent with earlier published findings demonstrating that apoAIF cannot be localized on the OMM [20].

**Fig. 3:**
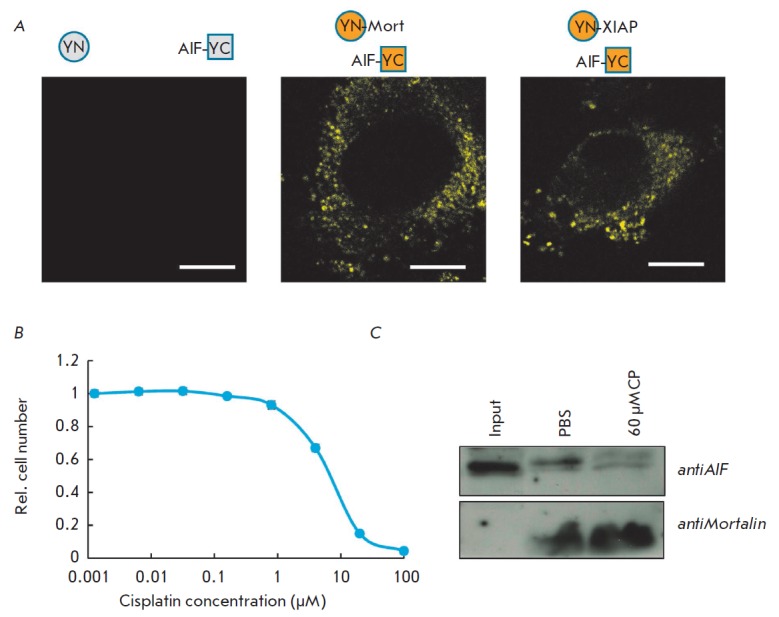
AIF interacts with mortalin in living cells. A – YFP fluorescence in U87-MG cells coexpressing YC-AIF and YN (left panel), YN-Mort (middle panel) or YN-XIAP (right panel). B – Survival of U87-MG cells treated with different concentrations of cisplatin. C – Western blotting for endogenous AIF that was eluted from magnetic beads with immobilized recombinant mortalin. U87-MG cells treated or untreated with 60 µM of cisplatin were lysed and cell lysate was incubated with magnetic beads with immobilized mortalin. Bounded proteins were eluted and separated in gel. Recombinant mortalin was detected as a loading control

Next, we used the biomolecular fluorescence complementation (BiFC) assay to confirm that AIF can interact with mortalin not only *in vitro *but also *in vivo *inside a living cell. This method relies on the fact that YFP protein retains its fluorescence properties even after it has divided into two parts, in case both parts are located close enough to one another. For this very reason, we constructed plasmids encoding mortalin bound to the N-terminal fragment of YFP (Mort-YN) and fAIF carrying the C-terminal fragment of YFP (YC-AIF) (*Fig. 3A*). These plasmids were used to co-transfect U87MG human glioblastoma cells. Furthermore, cells co-transfected with the plasmids encoding YC-AIF and XIAP protein with the N-terminal portion of YFP (YN-XIAP) served as the positive control. It has been demonstrated earlier that AIF interacts with XIAP in mitochondria [18]. Cells co-transfected with the plasmids encoding YC-AIF and the unbound N-terminal portion of YFP (YN) served as the negative control. Confocal microscopy images of cells co-expressing different pairs of proteins were obtained 24 h post-transfection. *Figure 3A* shows the bright fluorescence of YFP in cells co-expressing YC-AIF and Mort-YN, as well as in cells co-expressing YC-AIF and YN-XIAP. These findings confirm the hypothesis that AIF and mortalin interact with one another *in vivo*.

After obtaining the results demonstrating that AIF can interact with mortalin both *in vitro *and *in vivo*, we investigated alterations in the intensity of this interaction during apoptosis. For this reason, we first treated U87MG cells with cisplatin [Pt(NH_3_)_2_Cl_2_], a drug widely used in the chemotherapy of many types of tumors. In order to determine the cisplatin concentration that causes apoptosis in most cells, we evaluated the effect of various amounts of this compound on cell viability by staining cells with a Alamar blue dye. As shown in *Fig. 3B*, 60 μM cisplatin was enough to induce apoptosis in the majority of the cells. Therefore, the cells were treated with 60 μM cisplatin for 24 h and lysed. The lysate was incubated with magnetic beads with immobilized recombinant mortalin. Lysate of normal cells not treated with cisplatin was used as a control. After the incubation and subsequent washing, the bound proteins were eluted and subjected to polyacrylamide gel electrophoresis. AIF was detected by Western blotting with primary antibodies specific to this protein. *Figure 3C* demonstrates that recombinant mortalin interacts with endogenous full-length AIF in normal cells, while the intensity of this interaction decreases noticeably once apoptosis is induced.

**Fig. 4:**
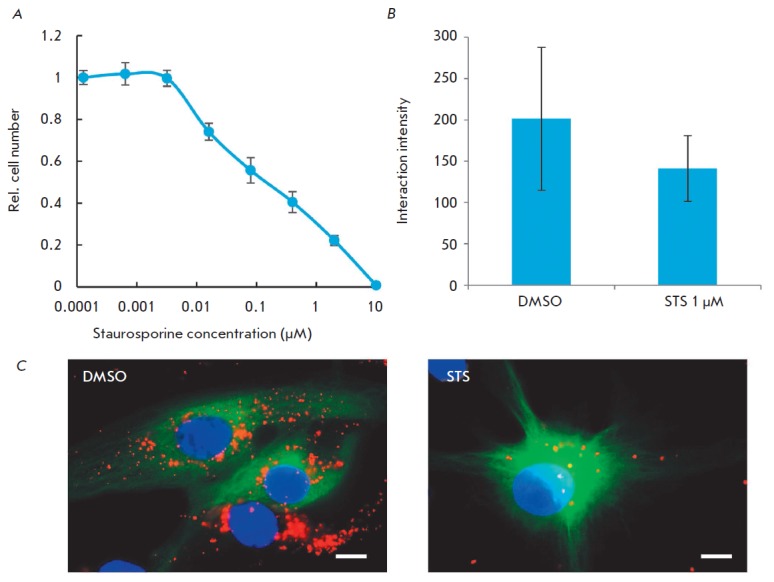
AIF interacts with mortalin in fixed cells. A – Survival of U87-MG cells treated with different concentrations of staurosporine. B – Effect of staurosporine on Mortalin-AIF interaction intensity determined by PLA assay in U87-MG cells (p < 0,05). C – Visualization of Mortalin-AIF interaction by PLA assay in U87-MG cells treated with 1 μM staurosporine (STS) or DMSO as a control

To further confirm our data, we performed proximity ligation assay (PLA) for mortalin and AIF using staurosporine, a widely known apoptosis inducer. During PLA, the fixed and permeabilized cells are incubated with two primary antibodies specific to the investigated proteins and a pair of oligonucleotide-conjugated secondary antibodies. If the oligonucleotides conjugated to different antibodies turn out to lie in appreciably close proximity to each other as happens during interaction of the target proteins, addition of DNA polymerase induces rolling circle DNA amplification on these nucleotides. Next, the cells are incubated with a fluorescently labeled oligonucleotide probe, which is annealed at the amplification sites of the respective DNA. Intracellular protein–protein interactions are eventually visualized as single-point fluorescent regions, with their number being proportional to the binding intensity of the analyzed proteins [23]. Similarly to the previous experiment, we measured the concentration of staurosporine causing apoptosis in most cells (*Fig. 4A*). Next, we used PLA to study the alterations in the intensity of interaction between endogenous AIF and mortalin after induction of apoptosis (*Fig. 4B,C*). Our findings indicate that in apoptotic cells the intensity of mortalin–AIF interactions is significantly reduced (*p * < 0.05), which agrees well with the data obtained in the previous experiment.

**Fig. 5:**
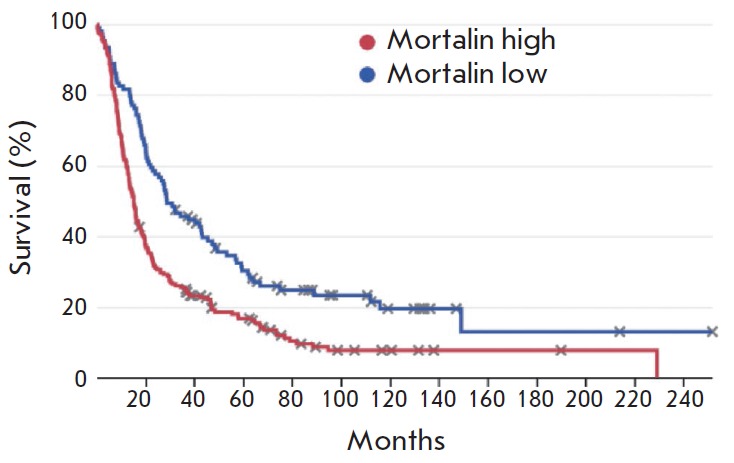
Kaplan-Meier survival curve for patients with glioma divided into two groups based on mortalin expression level. Results were obtained by bioinformatic analysis of the Rembrandt database. p = 0,00002 (log-rank t-test)

According to the results described above, it is fair to assume that the elevated mortalin level in cancer cells may increase the intensity of the binding between AIF and the OMM, thus being responsible for the increased apoptosis resistance of cells and, therefore, a more aggressive tumor phenotype. We tested a possible association between mortalin expression and the survival rate of patients using the open-access Rembrandt database containing data on gene expression and the survival rates of patients with brain tumors. An analysis of this database (*Fig. 5*) demonstrated that a higher level of mortalin expression statistically significantly correlates with poor survival of glioma patients.

## DISCUSSION

The caspase-independent apoptotic pathway consists of two stages. First, AIF is dissociated from the mitochondrial membrane. Next, cytoplasmic AIF is imported into the cell nucleus and activates DNA fragmentation. It is interesting that the second stage of this process (translocation of AIF into the nucleus) is inhibited by the Hsp70 protein, which is present in the cytoplasm and binds to the 150–228 a.a. of AIF [24]. We have demonstrated for the first time that another member of the family of heat shock proteins, mortalin, which binds to the 1–102 a.a. of AIF, is involved in the AIF–OMM interaction. Hence, it is fair to hypothesize that heat shock proteins are important inhibitors of the caspase-independent apoptotic pathway. Mortalin protein can anchor AIF to the OMM and impede apoptosis induction. However, if AIF is dissociated from the OMM, Hsp70 protein inhibits AIF translocation into the nucleus. This hypothesis agrees well with the recently published reports indicating that mortalin can hinder apoptosis by anchoring HIF1a, another essential protein that regulates cellular response to stress, to the outer surface of the OMM [21]. As proof for our findings, we demonstrated that elevated mortalin expression correlates with an aggressive phenotype of cancer and, therefore, is a poor prognostic factor for patients with brain tumors.

## CONCLUSIONS

Although many studies have focused on the caspase-independent apoptotic pathway, the first and most important stage of this process (namely, AIF dissociation from the outer mitochondrial membrane) is still a riddle to be solved. Our findings demonstrate that mortalin protein is involved in the binding between AIF and the OMM. Additional experiments are needed to evaluate the contribution of this interaction to the localization of AIF to the OMM. However, our findings and the data published earlier provide grounds for assuming that when a cell is exposed to DNA-damaging agents, poly-ADP-ribose is produced in the nucleus and further translocated into the cytoplasm, where it binds to AIF [8, 16]. This interaction alters the conformation of AIF and makes it lose its ability to bind to mortalin, through which AIF is anchored to the OMM. As a result, AIF is dissociated from the OMM and translocated into the nucleus, where it causes a cascade of events, eventually leading to cell death. However, further research is needed to confirm the interaction between endogenous AIF and mortalin and to prove that specific inhibition of binding between these proteins will cause dissociation of AIF from the OMM and eventual cell death.

## Abbreviations

AIF: apotosis inducing factor
Hsp: heat-shock proteins
OMM: outer mitochondrial membrane
BiFC: bimolecular fluorescence complementation
YFP: yellow fluorescent protein
PLA: in situproximity ligation assay

## References

[1] Reed J.C. (1999). J Clin Oncol..

[2] Kühtreiber W.M., Hayashi T., Dale E.A., Faustman D.L. (2003). J. Mol. Endocrinol..

[3] Cao G., Xing J., Xiao X., Liou A.K., Gao Y., Yin X.M., Clark R.S., Graham S.H., Chen J. (2007). J. Neurosci..

[4] Liu T., Biddle D., Hanks A.N., Brouha B., Yan H., Lee R.M., Leachman S.A., Grossman D. (2006). J. Invest. Dermatol..

[5] Mahmud H., Dälken B., Wels W.S. (2009). Mol. Cancer Ther..

[6] Galluzzi L., Joza N., Tasdemir E., Maiuri M.C., Hengartner M., Abrams J.M., Tavernarakis N., Penninger J., Madeo F., Kroemer G. (2008). Cell Death Differ..

[7] Choudhury S., Bae S., Ke Q., Lee J.Y., Kim J., Kang P.M. (2011). Basic Res. Cardiol..

[8] Wang Y., Kim N.S., Haince J.F., Kang H.C., David K.K., Andrabi S.A., Poirier G.G., Dawson V.L., Dawson T.M. (2011). Sci. Signal..

[9] Cho B.B., Toledo-Pereyra L.H. (2008). J. Invest. Surg..

[10] Norberg E., Orrenius S., Zhivotovsky B. (2010). Biochem. Biophys. Res. Commun..

[11] Lorenzo H.K., Susin S.A., Penninger J., Kroemer G. (1999). Cell Death Differ..

[12] Brown D., Yu B.D., Joza N., Bénit P., Meneses J., Firpo M., Rustin P., Penninger J.M., Martin G.R. (2006). Proc. Natl. Acad. Sci. U S A..

[13] Baritaud M., Boujrad H., Lorenzo H.K., Krantic S., Susin S.A. (2010). Cell Cycle..

[14] Arnoult D., Parone P., Martinou J.C., Antonsson B., Estaquier J., Ameisen J.C. (2002). J. Cell. Biol..

[15] Yu S.W., Wang H., Poitras M.F., Coombs C., Bowers W.J., Federoff H.J., Poirier G.G., Dawson T.M., Dawson V.L. (2002). Science..

[16] Yu S.W., Wang H., Dawson T.M., Dawson V.L. (2003). Neurobiol. Dis..

[17] Parsons D.F., Williams G.R., Chance B. (1966). Ann. N.Y. Acad. Sci..

[18] Wilkinson J.C., Wilkinson A.S., Galbán S., Csomos R.A., Duckett C.S. (2008). Mol. Cell. Biol..

[19] Pavlyukov M.S., Antipova N.V., Balashova M.V., Vinogradova T.V., Kopantzev E.P., Shakhparonov M.I. (2011). J. Biol. Chem..

[20] Yu S.W., Wang Y., Frydenlund D.S., Ottersen O.P., Dawson V.L., Dawson T.M. (2009). ASN Neuro..

[21] Mylonis I., Kourti M., Samiotaki M., Panayotou G., Simos G. (2017). J. Cell. Sci..

[22] Londono C., Osorio C., Gama V., Alzate O. (2012). Biomolecules..

[23] Maszczak-Seneczko D., Sosicka P., Olczak T., Olczak M. (2016). Methods Mol. Biol..

[24] Gurbuxani S., Schmitt E., Cande C., Parcellier A., Hammann A., Daugas E., Kouranti I., Spahr C., Pance A., Kroemer G. (2003). Oncogene..

